# Mucocutaneous manifestations in juvenile-onset systemic lupus erythematosus: a review of literature

**DOI:** 10.1186/1546-0096-13-1

**Published:** 2015-01-05

**Authors:** Direkrit Chiewchengchol, Ruth Murphy, Steven W Edwards, Michael W Beresford

**Affiliations:** Institute of Translational Medicine, Alder Hey Children’s NHS Foundation Trust, University of Liverpool, Liverpool, UK; Institute of Integrative Biology, University of Liverpool, Liverpool, UK; Immunology Unit & Center of Excellence in Immunology and Immune-mediated Disease, Department of Microbiology, Faculty of Medicine, Chulalongkorn University, Bangkok, Thailand; Department of Dermatology, Queens Medical Centre, Nottingham University Teaching Hospitals, Nottingham, UK; Department of Women’s and Children’s Health, Institute of Translational Medicine, Alder Hey Children’s NHS Foundation Trust, Eaton Road, Liverpool, L12 2AP UK

**Keywords:** Juvenile-onset systemic lupus erythematosus, Mucocutaneous lupus lesions, Lupus erythematosus specific lesions, Lupus erythematosus nonspecific lesions, Diagnosis, Treatment

## Abstract

Patients diagnosed with juvenile-onset systemic lupus erythematosus (JSLE) often have skin and oral lesions as part of their presentation. These mucocutaneous lesions, as defined by the American College of Rheumatology (ACR) in 1997, include malar rash, discoid rash, photosensitivity and oral ulcers. It is therefore essential to recognize mucocutaneous lesions to accurately diagnose JSLE. The mucocutaneous lesions can be divided into those with classical histological features (LE specific) and those strongly associated with and forming part of the diagnostic spectrum, but without the classical histological changes of lupus (LE nonspecific). A malar rash is the most commonly associated LE specific dermatological presentation. This skin manifestation is an acute form and also correlates with disease activity. Subacute (polycyclic or papulosquamous lesions) and chronic (discoid lesions) forms, whilst showing classical histological changes supportive of lupus, are less commonly associated with systemic lupus and do not correlate with disease activity. The most commonly associated skin lesions without classical lupus changes are cutaneous vasculitis, oral ulcers and diffuse non-scarring alopecia. These signs frequently relate to disease activity. An understanding of cutaneous signs and symptoms of lupus in children is important to avoid delay in diagnosis. They will often improve as lupus is adequately controlled and their reappearance is often the first indicator of a disease flare.

## Introduction

Juvenile-onset systemic lupus erythematosus (JSLE) is one of the most common systemic autoimmune connective tissue disorders in children. The disease severity varies from mild to severe, and requires long term and often aggressive treatment. It is estimated that 15-20% of SLE patients develop signs and symptoms during childhood and adolescence [1–3].

The incidence of JSLE across the world varies between 0.3 to 0.9 per 100,000 per year with estimated prevalence between 0.3 to 8.8 per 100,000 [4–7]. Females are more likely to be affected (male and female ratio; 1:3 to 1:5) with the peak age of presentation around puberty (median age onset 12.1) [8–10]. The clinical presentation of JSLE is frequently more severe than adult onset SLE with multiple organ involvement, particularly the kidney and central nervous system [11–18].

The diagnosis of JSLE is made in accordance with the American College of Rheumatology classification (revised criteria 1997) [19], although recent modifications to these criteria have been proposed [20]. The ACR criteria include several mucocutaneous manifestations: malar (butterfly) rash; discoid rash; photosensitivity and oral ulcers. Patients will often present with skin and oral lesions as initial clinical manifestations of the disease. It is therefore important to recognize these mucocutaneous manifestations of the disease.

In adults, it is common to have limited manifestations of lupus affecting just the skin without fulfilling the ACR diagnostic criteria. This is in contrast, to lupus skin lesions in children which are usually associated with systemic involvement [21]. This article focuses on the mucocutaneous aspects of JSLE.

## Review

### Epidemiology

Mucocutaneous manifestations are very common in both adult and JSLE (60-85%) [22]. When considering the ACR diagnostic criteria, dermatological manifestations occur second to involvement of the hematological (50-100%) and musculoskeletal systems (60-90%) [22, 23]. The most common mucocutaneous lesions in JSLE are: malar rash, photosensitivity, cutaneous vasculitis and oral or nasal ulcers. Others include generalized lupus rash, non-scarring alopecia, livedo reticularis and Raynaud’s phenomenon. Although the prevalence of mucocutaneous manifestations in paediatric and adult patients are quite similar, some lesions are clearly less common in children; such as subacute cutaneous lesions, a discoid rash and livedo reticularis [2, 23–28]. Table 1 summarizes the comparative frequency of mucocutaneous manifestations in JSLE and adult SLE. In JSLE, lupus specific mucocutaneous manifestations appear more frequently [29].

**Table 1:** **Comparative frequency of mucocutaneous lesions in juvenile systemic lupus erythematosus (JSLE) and adult systemic lupus erythematosus (adult SLE)**

Mucocutaneous lesions	JSLE	Adult SLE
**1.** ***LE specific skin lesions***		
Malar rash	44-85% [2, 5, 6]	40-52% [5, 6]
Generalized lupus rash	30% [28]	N/A [5, 6]
Subacute cutaneous LE	Rare [8, 33, 34]	7-27% [8, 9]
Discoid rash	<10% [1, 2, 4, 18, 28, 29]	20-50% [5, 36]
Generalized DLE	10-37% [31, 34–36]	40-49% [36]
Lupus panniculitis/ profundus	<1% [34, 37]	1-3% [10]
**2.** ***LE nonspecific skin lesions***		
Cutaneous vasculitis	16-45% [11, 21, 47]	11-70% [5, 7, 12, 13]
Photosensitivity	35-50% [2, 4, 5]	63% [7]
Oral and nasal ulcers	20-40% [2, 4, 5, 49, 52]	18-30% [6, 7, 49, 52]
Non-scarring alopecia	15-30% [2, 5]	25-55% [2, 5, 53]
Livedo reticularis	6-12% [14, 30, 33]	22-35% [12]
Raynaud’s phenomenon	6-12% [15, 16]	10-45% [15, 16, 60, 61]
Bullous SLE	<1% [28]	N/A [62–64]

N/A; A lack of published evidence for the relative frequency of these lesions.

### Classifications and clinical manifestation

The classifications of mucocutaneous manifestations were first developed in the 1970s [27] and are divided into 2 categories; lupus erythematosus specific skin lesions (LE specific), and nonspecific skin lesions (LE nonspecific). This classification is used in both juvenile and adult onset forms of the disease and most of these appear similarly in both age groups. This review will highlight any differences between the two age groups.

#### Specific mucocutaneous lesions

These lesions are categorized into 3 forms.

**Figure 1:**
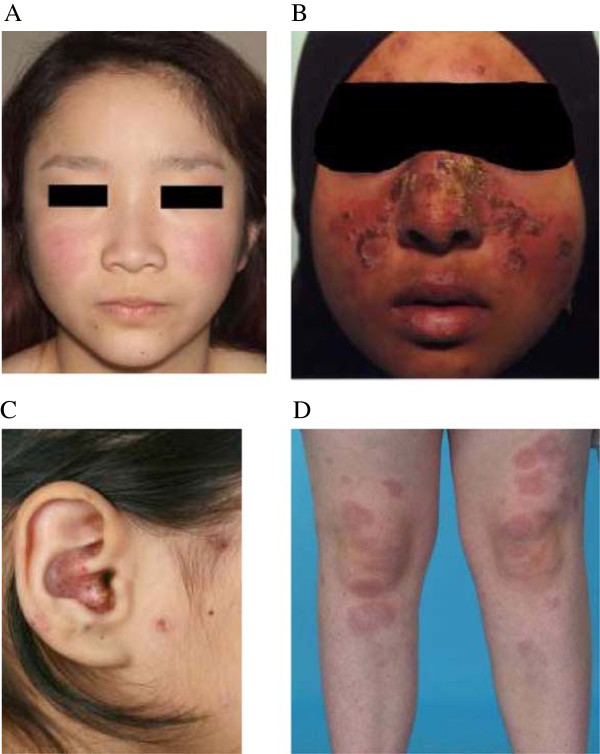
**LE specific skin lesions: (A) Malar (butterfly) rash (mild symptom); (B) Malar rash with interface dermatitis; (C) Crusting and interface changes affecting the ear; and (D) Generalized ACLE or maculopapular lupus rash at both knees.**

**Acute cutaneous lupus erythematosus (ACLE)** ACLE presents with localized or generalized lesions and they are very sensitive to ultraviolet light. Localized ACLE or malar (butterfly) rash is the most common LE specific lesion in both JSLE and adult SLE patients [23, 30, 31]. It is characterized by a well defined, symmetrical erythematous and edematous, non-pruritic malar rash, over the nasal bridge and typically sparing the nasolabial folds (Figure 1A and B). Lesions may also involve the ears and may mimick an interface dermatitis (Figure 1C). They typically resolve with post-inflammatory hypo/hyperpigmentation. Less frequently, lesions in ACLE are a more diffuse rash involving non-light exposed sites, often with extensive erythema and edema (Figure 1D). Both the localized malar rash and the more diffuse erythema are strongly related to systemic disease activity in JSLE and adult SLE [23, 30, 31].

**Subacute cutaneous lupus erythematosus (SCLE)** SCLE is extremely rare in JSLE patients [32], but more common in adult SLE patients. There are two forms: annular/polycyclic lesions and papulosquamous/psoriasiform lesions in adults and both forms are found in JSLE patients. The lesions are characterized as widespread, symmetrical, erythematous papules/plaques, with scales and telangiectasia on sun-exposed and non-sun-exposed areas, such as the chest and back. They are particularly common on the face and upper extremities and usually heal without scaring [32]. Skin involvement in of the lower extremities is rare in adults but is more common in children [33].

Numerous cases of drug-induced SCLE have been reported in adults such as antihypertensive drugs, anticonvulsants and antihistamines [29]. As the clinical features of drug-induced SCLE lesions cannot be reliably differentiated from SCLE lesions, any suspected drugs should be discontinued but these are seldom used in childhood.

**Chronic cutaneous lupus erythematosus (CCLE)** In adults, it is common to see discoid lupus on the skin without underlying systemic involvement. This is a rare presentation in children particularly below the age of 10 [12, 22, 23, 26]. Discoid lupus has rarely been reported in children without systemic symptoms [28, 33, 34]. These lesions occur most commonly on the scalp (vertex), face and ears. The lesions usually occur above the neck as scarring indurated, purplish papules, expanding into coin-shapes with atrophic formation and telangiectasia. If lesions are on the scalp, permanent hair loss ensues. Follicular plugging usually occurs because an adherent scale becomes stuck to the hair follicles. Interestingly, the risk of progression from DLE to SLE is much higher in children than in adults (23.5-26% VS 5-10%). The risk seems to be greater in children with a family history of autoimmune rheumatic disease [28, 34]. Generalized DLE (above and below the neck) in children is described in several studies and appears to be associated with a worse prognosis [28, 33–35].

Other forms of CCLE are rare in children, such as lupus panniculitis and lupus profundus [33, 36], mucosal LE and chilblain LE [37–40], and tumid LE [41–43]. Lupus erythematosus/lichen planus overlap syndrome has been only reported in adults [44, 45].

#### Nonspecific mucocutaneous lesions

The key difference between these nonspecific skin manifestations in lupus compared to the lupus specific manifestations, is that they appear not only in JSLE, but also in other inflammatory diseases. Most LE nonspecific lesions commonly found in children and adults are similar and both affect vasculature (e.g. cutaneous vasculitis, livedo reticularis and Raynaud’s phenomenon). The other common lesions are photosensitivity, oral ulcers and diffuse non-scarring alopecia.

**Figure 2:**
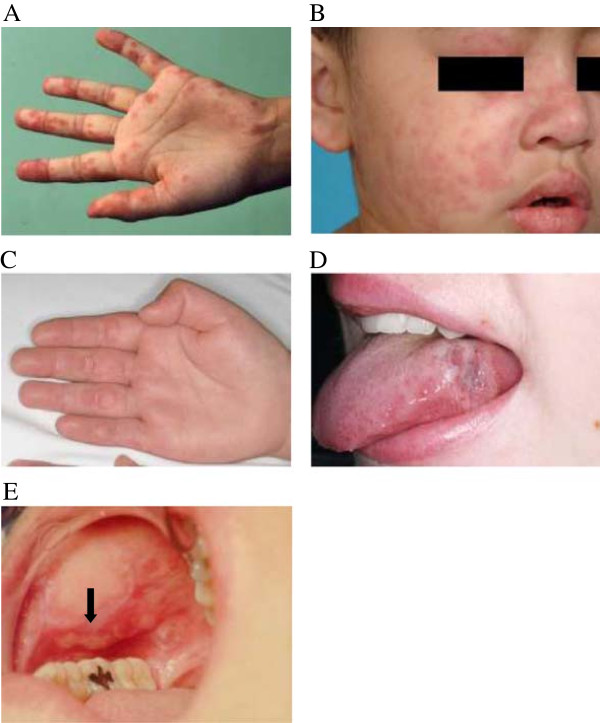
**LE nonspecific skin lesions: (A) Vasculitic purpura at left palm; (B) Cutaneous vasculitis at right cheek, eyelid and nose; (C) Cutaneous urticarial vasculitis at right palm; (D) Oral discoid lesion on the lateral border of the tongue; and (E) Oral ulceration and a discoid lesion on the hard palate.**

**Cutaneous vasculitis** Cutaneous vasculitis usually affects small blood vessels (leukocytoclastic vasculitis). The lesions are characterized as petechiae or palpable purpura (Figure 2A), and may occasionally blister. They are commonly found on the face, palms and soles of the feet (Figure 2B). The lesions are induced by the formation of immune complexes and neutrophilic infiltration, and the presence of vasculitic lesions strongly relates to systemic disease activity [46]. Other clinical presentations include: punctate lesions and urticarial vasculitis (Figure 2C).

**Photosensitivity** Photosensitivity can appear as any skin rash reacting to both UVA and UVB light. Diagnosis is based on patient history or clinician observations. The lesion occurs on sun-exposed areas (such as the face, upper chest, or extremities) and becomes progressively worse after sun-exposure. Although the lesions usually develop during active disease, it is unknown if they correlate with systemic disease. Photosensitivity with malar rash is commonly found in juvenile dermatomyositis; thus, other lupus features are needed in order to differentiate between the two conditions [47].

**Oral or nasopharyngeal ulcers** There are two types of these ulcers: those with classical LE histological changes representing oral discoid lesions (Figure 2D) and nonspecific ulcers in keeping with aphthous ulceration [48]. The lupus specific lesions begin with solitary erythema and hemorrhaging patches before developing into discoid ulcers with a reticulate border. Typically, the lesions are painless and located on the hard palate (Figure 2E). In contrast, the nonspecific aphthous ulcers are usually painful, with multiple lesions on the buccal mucosa, lips and nasal septum, whilst also having a tendency to bleed [49, 50]. Oral and nasopharyngeal ulcers are normally found during active disease and subside with disease remission in JSLE and adult SLE patients [30, 48, 51].

**Diffuse non-scarring alopecia** Alopecia often presents with generalized hair loss without signs of inflammation on the scalp. Diffuse, non-scarring alopecia in JSLE and adult SLE patients usually suggests active disease [52]. However, it may occur three months after a severe lupus flare; e.g. Telogen effluvium, which is a nonspecific finding and can also occur after any significant systemic disturbance [53]. Other forms of alopecia also found in JSLE patients include lupus hair (thin and weakened hair at the periphery of the scalp), patchy non-scarring alopecia (mild erythematous, scattered patchy hair loss) and alopecia areata [54].

**Livedo reticularis** These lesions present more commonly in both juvenile and adult patients who are diagnosed with anti-phospholipid syndrome [55]. It is characterized by erythematous or cyanotic discoloration of the skin with reticulated (net-like) pattern, usually on the lower extremities. The etiology and correlation with systemic disease activity are unknown, but vascular obstruction and blood viscosity may be the cause [56].

**Raynaud’s phenomenon** Raynaud’s phenomenon is characterized by the classic "triphasic" color changes limited to the digits; pallor (white or blanching) followed by the cyanosis (blue) then erythema (red or reactive hyperemia). This color change sequence results from excessive vasospasms triggered by cold exposure or emotional stress, and can often be reversed following re-warming. There is no evidence to suggest that this symptom correlates with systemic disease [57, 58].

**Bullous SLE** The bullous lesions are rare and most commonly reported in young adults and African-American women [59]. It is characterized by multiple, tense vesicles/bullae usually on face, neck and upper trunk. As bullous SLE is similar to other vesicobullous conditions, the criteria needed to make a diagnosis are: (a) acquired widespread cutaneous vesicobullous lesions; (b) subepidermal blister with acute neutrophilic infiltration in dermis confirmed by histopathology; (c) evidence of IgG at the dermal side of the basement membrane zone by direct or indirect immunofluorescence; (d) presence of antibodies to collagen type VII by indirect immunofluorescence on salt-split skin and (e) a tendency to respond to Dapsone [60]. There is no evidence of any correlation between bullous SLE and systemic disease activity [61].

**Other LE nonspecific lesions** Other lesions include: calcinosis cutis, acanthosis nigricans, hypocomplementaemic urticarial vasculitis (very rare in JSLE patients) [62–64], whilst rheumatoid nodules, anetoderma and erythromelalgia have been reported in adult SLE [65–67].

### Diagnosis of cutaneous lupus

Careful clinical assessment at presentation is usually sufficient to make a diagnosis of mucocutaneous lupus lesions. Skin histopathology may be useful, but this needs to be carefully considered in children because of scarring after this procedure. Laboratory investigations in JSLE based on the ACR criteria can also guide and support the diagnosis of the affected lesions in patients diagnosed with JSLE. Moreover, some of these findings are more commonly associated with particular lesions; for example, anti-Ro/SSA is often detected in JSLE patients presenting with SCLE [32].

#### Histopathology

Histopathological findings of the affected lesions of JSLE and adult SLE usually show similarities. Characteristically, skin histopathology of a LE specific lesion is interface dermatitis with basement membrane damage. Mild to severe inflammation is present in the dermis and skin appendages with perivascular lymphocytic and neutrophil infiltrates called leukocytoclastic vasculitis [68–70]. Abundant extracellular deposition of mucin is markedly observed in the dermis between the collagen bundles and sweat glands [68–70]. In particular, skin histopathology of DLE shows classical features: vacuolar degeneration of basal cell layer; thickened basement membrane; orthokeratosis with follicular plugging in upper dermis. Epidermal atrophy with marked thickening of basement membrane occurs in the later stages. Dermal fibrosis and follicular atrophy with abundant extracellular mucin deposits between collagen bundles in reticular dermis and sweat glands are eventually seen [68–70].

The histopathology features attributed to nonspecific lupus vary depending on the type of lesions and are not pathognomonic of the condition. For example, cutaneous vasculitis in JSLE shows small vessel leukocytoclastic vasculitis including endothelial cell damage, fibrin deposition, inflammatory cell infiltrate (predominantly neutrophils) and nuclear dust. These findings can be found in juvenile patients with other systemic diseases such as henoch-schönlein purpura, juvenile dermatomyositis and systemic juvenile idiopathic arthritis [71].

#### Immunofluorescence findings

Immunofluorescence is not routinely used for the diagnosis in JSLE patients with mucocutaneous lesions, but it can be helpful when supporting evidence for making a diagnosis is needed. Direct immunofluorescence shows band-like deposits of IgG, IgM and complement factor (C3) in the basement membrane zone or dermo-epidermal junction (lesional lupus band test) [72]. However, false positive findings can occur especially in sun-exposed areas. Immune deposits are also found in non-lesional skin biopsies (non-lesional lupus band test).

### Management

Careful assessment of systemic disease status is first needed, as inducing disease remission will lead to resolution of the mucocutaneous lesions. Treatment is tailored to disease severity and organ involvement. In limited cutaneous disease, topical corticosteroids may be helpful. In more extensive disease or where there is systemic involvement, short courses of systemic corticosteroids with concurrent use of hydroxychloroquine and/or immunosuppressive therapies are indicated. Table 2 summarizes the commonly used treatments of mucocutaneous lesions and their common side effects in JSLE patients.

**Table 2:** **Summary of commonly used treatment in mucocutaneous lupus lesions in juvenile systemic lupus erythematosus (JSLE)** [74–81]

Treatments	Dose	Indications	Common/serious side effects
**1. Topical**			
***Sunscreen***	2 mg/cm^2^, SPF >30	All sun-exposure areas apply at least 30 min before sun exposure	Greasy and allergic contact dermatitis
***Topical steroids***			
- *Mild potency*	1% hydrocortisone acetate	Eyelids, face and intertriginous areas	Hypopigmentation, skin atrophy, increased hair growth and telangiectasia;
- *Moderate potency*	0.1% triamcinolone acetonide	Scalp and body	
	0.1% mometasone furoate		
- *High potency*	0.05% clobetasone propionate	Scalp, palms and soles	
	0.05% betamethasone dipropionate		
***Intralesional steroids***	2.5-10 mg/mL	Discoid lesions particularly on scalp	Skin atrophy and hypopigmentation
***Calcineurin inhibitors***	1% pimecrolimus	Eyelids, face and intertriginous areas (steroid-sparing effects)	Burning sensation and infection
	0.03%, 0.1% tacrolimus		
**2. Systemic**			
***Systemic Steroids***	0.5-2 mg/kg ideal body weight per day between 2–4 weeks, followed by tapering dose	Severe skin lesions or systemic disease flare up	Osteoporosis, cushing syndrome and growth retardation
***Hydroxychloroquine***			
- *Children*	5 mg/kg ideal body weight per day	Combination with systemic steroids	Ocular toxicity, gastrointestinal upset, dizziness and headache
- *Young adults*	6-6.5 mg/kg ideal body weight per day	(steroid-sparing effects)	

#### Sunscreen

Mucocutaneous lupus lesions are generally highly photosensitive, triggered by both UVA and UVB [73]. Children are advised to avoid prolonged sun exposure and routinely wear protective clothing (including hats). Very potent physical and chemical sunscreens include: titanium dioxide, zinc oxide, tocopheryl acetate and flavonoids. These can be used in children, particularly before direct sun exposure. Children in tropical countries may have some difficulties of sun avoidance; thus, parents as well as school teachers are encouraged to be vigilant about UV protection. Lack of sun exposure in affected individuals can sometimes result in low vitamin D levels; if so, vitamin D3 and calcium supplements should be considered [74].

#### Corticosteroids

Topical corticosteroids are commonly used in JSLE patients as they are very effective for mucocutaneous lupus lesions [74]. High potency corticosteroids are necessary to induce remission of the lesions. This can then be followed by gradual tapering of the dose to discontinuation. Mild to moderate potency corticosteroids are usually applied to lesions on the face and body. However, calcineurin inhibitors are preferred in delicate areas (e.g. around the eyes, groin and genitalia) for more prolonged use to prevent skin thinning. If the lesions are part of JSLE, then they will only be improved along with the systemic therapies used to control the disease. In this group, treatments should be more focused on controlling the systemic disease.

An intralesional corticosteroid injection can be used in adolescents with DLE, particularly on the scalp, to minimize the scarring alopecia [75], whilst systemic corticosteroids are very helpful in the short term of induce remission of severe mucocutaneous lesions (e.g. multiple oral ulcers, or severe cutaneous vasculitis with ulceration and necrosis). However, these should be avoided for prolonged periods due to serious side effects [76]. Therefore, the affected areas, the route of administration, the potency of corticosteroids, length of treatment and any side effects should be carefully considered, particularly in JSLE patients.

#### Aminoquinolone antimalarial drugs

Hydroxychloroquine demonstrates good efficacy with mucocutaneous lupus lesions and is therefore the first line treatment when systemic therapy is required [21]. The mechanism of action of these drugs is uncertain but includes Toll-Like Receptor blockade that prevent antigen stimulation, a process thought to be involved in the pathogenesis of the disease [77]. Serious side effects in JSLE, particularly irreversible ocular damage, require careful consideration as they depend on the maximum daily dose rather than the cumulative dose. Therefore, using the ideal body weight for calculation of the daily dose in children is important [78]. Routine eye check-ups is recommended.

#### Other medications

Topical calcineurin inhibitors, such as tacrolimus and primecrolimus, are useful for lesions that are particularly sensitive to topical corticosteroids and prone to skin atrophy, such as on the eyelids, face and intertriginous areas and are well-tolerated [79–81]. Steroid sparing immunosuppressive agents in JSLE patients (e.g. methotrexate, azathioprine and mycophenolate mofetil), are usually initiated along with hydroxychloroquine [74].

## Conclusion

Patients with JSLE commonly present with mucocutaneous manifestations, and it is therefore important to recognize the lesions to make an accurate diagnosis. Assessment of systemic disease status in any child presenting with mucocutaneous lupus features is crucial as it may take a number of years to meet the diagnostic criteria. Therefore, any child with mucocutaneous lesions associated with SLE needs to be regularly reassessed and monitored. Sun protection is vital and should be encouraged to prevent both worsening of the symptoms and exacerbation of SLE. In adult patients, lupus associated skin disease is often localized. In children, lesions are usually associated with systemic disease and require treatment with systemic immunosuppressive drugs in order to achieve adequate disease control.

### Consent

Written informed assent/consent forms were received from all patients and their parents. This research received no specific grant from any funding agency in the public, commercial, or not-for-profit sectors.
